# C-reactive protein/albumin ratio is a prognostic indicator for predicting surgical intervention and mortality in neonates with necrotizing enterocolitis

**DOI:** 10.1007/s00383-021-04879-1

**Published:** 2021-03-29

**Authors:** Amir T. Mohd Amin, Rafdzah A. Zaki, Florian Friedmacher, Shazia P. Sharif

**Affiliations:** 1 Department of Pediatric Surgery, The Royal London Hospital, London, UK; 2 Centre for Epidemiology and Evidence Based Practice, Department of Social and Preventive Medicine, Faculty of Medicine, University of Malaya, Kuala Lumpur, Malaysia; 3 Department of Pediatric Surgery, University Hospital Frankfurt, Goethe University Frankfurt, Theodor-Stern-Kai 7, 60590 Frankfurt, Germany

**Keywords:** Necrotizing enterocolitis, C-reactive protein, Albumin, Surgery, Mortality, Outcomes

## Abstract

**Purpose:**

The role of hypoalbuminemia and raised C-reactive protein (CRP) levels in predicting critical prognosis has been described extensively in adult literature. However, there are limited studies in pediatrics, particularly neonates. The CRP/albumin (CRP/ALB) ratio is often associated with higher mortality, organ failure and prolonged hospital stay. We hypothesized that the serum CRP/ALB ratio has a prognostic value in predicting surgery and mortality in neonates with necrotizing enterocolitis (NEC).

**Methods:**

Retrospective review of all neonates with clinical and radiological evidence of non-perforated NEC that were treated in a tertiary-level referral hospital between 2009 and 2018. General patient demographics, laboratory parameters and outcomes were recorded. Receiver operating characteristics analysis was performed to evaluated optimal cut-offs and area under the curve (AUC) with 95% confidence intervals (CI).

**Results:**

A total of 191 neonates were identified. Of these, 103 (53.9%) were born at ≤ 28 weeks of gestation and 101 (52.9%) had a birth weight of ≤ 1000 g. Eighty-four (44.0%) patients underwent surgical intervention for NEC. The overall survival rate was 161/191 (84.3%). A CRP/ALB ratio of ≥ 3 on day 2 of NEC diagnosis was associated with a statistically significant higher likelihood for surgery [AUC 0.71 (95% CI 0.63–0.79); *p* < 0.0001] and mortality [AUC 0.66 (95% CI 0.54–0.77); *p* = 0.0150], respectively.

**Conclusions:**

A CRP/ALB ratio of ≥ 3 on day 2 is indicative of a critical pathway in neonates with radiologically confirmed, non-perforated NEC. This could be used as an additional criterion to guide parental counselling in NEC for surgical intervention and mortality.

## Introduction

Necrotizing enterocolitis (NEC) is one of the most common and critical gastrointestinal emergencies in neonates, which predominately affects premature and low birth weight infants in the first weeks of life. Surgical NEC is known to be associated with a significantly higher morbidity and mortality [1, 2]. However, the optimal timing for surgical intervention in neonates with NEC is not yet established [3, 4]. It has recently been shown that the largest proportion of perioperative deaths in pediatric surgery is secondary to NEC, with a 30-day perioperative mortality of up to 19% [5]. Hence, there is a need to identify validated early predictors for surgical intervention and mortality in neonates with NEC. Early predictors for surgery in neonates with NEC would optimize referral and treatment pathways, and potentially lead to improved outcomes [6]. Improving triage of neonates with NEC in non-surgical neonatal intensive care units in turn may lead to earlier referral, transfer and surgery. Ideally, a prognostic tool may predict surgical intervention in neonates with NEC as well as indicate the potential risk of death. This would also change parental counselling early in the disease process, thus enabling conversations to be tailored to each patient when discussing the probability of surgery and death in NEC cases [7].

The role of reduced serum albumin (ALB) and raised C-reactive protein (CRP) levels in predicting a critical prognosis has been described extensively in adult literature but is very limited in pediatrics. Research in adults has revealed that the CRP/ALB ratio is associated with higher mortality, organ failure and prolonged hospital stay in seriously ill or septic patients on acute medical wards and intensive care units, respectively [8, 9]. More recently, it has been demonstrated that a high CRP/ALB ratio is indicative of worse outcome (i.e. organ failure and/or death) in critically ill children on a pediatric intensive care unit [10]. The aim of this study was to investigate if the serum CRP/ALB ratio has a prognostic value in predicting surgical intervention and mortality in neonates with NEC.

## Materials and methods

### Study population and design

A retrospective cohort study of all neonates diagnosed with radiologically confirmed, non-perforated NEC that were consecutively admitted to the specialized neonatal intensive care unit at the Royal London Hospital (i.e. large, tertiary-level referral hospital) from 1 January 2009 to 31 December 2018 was performed. Details of each patient were identified using the BadgerNet platform (Clevermed, Edinburgh, United Kingdom)—a national electronic neonatal database. General patient demographics were collected on gender, pregnancy, gestational age and birth weight. Clinical presentation and examination findings were correlated with abdominal radiographs to ensure the patient met the inclusion criteria. Inclusion criteria were radiological evidence of NEC (i.e. pneumatosis intestinalis) and presence of one or more clinical findings (i.e. abdominal distension, bilious/bloody aspirates, blood per rectum, abdominal tenderness, abdominal wall erythema/discoloration or abdominal mass). The first day of NEC diagnosis was classified as day 1 of the disease process, and daily measured serum CRP and ALB levels were extracted from the individual electronical or paper medical records. When several blood samples were taken per day, the time of the first set of bloods was used and results from the next nearest tests done at 24 h, 48 h, etc. were recorded. If applicable, date of surgery and mortality data were noted for each patient. Indications for surgery were subsequent evidence of perforation, increasing abdominal distension, erythema, discoloration and tenderness despite maximal medical management and increasing ventilatory/inotropic support. Neonates presenting with radiologically confirmed perforated NEC were excluded from the study, which was formally registered with the clinical effectiveness unit at the Barts Health NHS Trust. As the study was retrospective in nature and therefore did not change the clinical management of patients involved, it was exempt from full IRB review but followed the principles expressed in the Declaration of Helsinki.

### Statistical analysis

Data was collated into an electronic spreadsheet and statistical analysis was performed using PASW Statistics 18.0 software application (SPSS Inc., Chicago, USA). Statistical differences between two cohorts of neonates with NEC (i.e. surgical intervention *vs.* conservative medical management) as well as outcome were analyzed using Chi-square or Fisher’s exact test, where appropriate. The serum CRP/ALB ratio on day 1, 2 and 3 of NEC diagnosis was determined in each patient. Bivariate and receiver operating characteristics (ROC) analysis was used to assess optimal cut-offs for the CRP/ALB ratio with sensitivity/specificity and to calculate the area under the ROC curves (AUC) with 95% confidence intervals (CI), respectively. A *p* value of < 0.05 was considered statistically significant.

## Results

### General patients’ demographics

A total of 191 consecutive neonates with radiologically confirmed, non-perforated NEC were identified during the study period. Of these, 106 (55.5%) were male and 85 (44.5%) were female. Median gestational age was 28.2 weeks (range 23.1–39.0) with a median birth weight of 1113 g (range 490–4140). One hundred and three (53.9%) were born at ≤ 28 weeks of gestation and 101 (52.9%) had a birth weight of ≤ 1000 g. In total, 84 (44.0%) patients eventually underwent surgical intervention for NEC. Indications for surgery were increasing abdominal distension, erythema or discoloration with tenderness despite maximal medical management and increasing ventilatory and/or inotropic support. Six patients had surgical intervention for a subsequent bowel perforation on a median of 3.5 days (range 1–10) after NEC diagnosis. Overall, for all neonates who underwent surgery, this occurred on a median of 3 days (range 1–23) of medical treatment. Except for gender and gestational age, there were no statistical difference between patients that had surgical intervention and those that did not (Table 1). The overall survival rate was 161/191 (84.3%). Of the 30 cases that died, 23 (76.7%) had surgery, compared to 61/161 (37.9%) of the surviving patients (*p* < 0.0001). Neonates who died were significantly more likely to be very premature (i.e. ≤ 28 weeks) and extremely low birth weight (i.e. ≤ 1000 g).

**Table 1:** General patients’ demographics and outcomes in 191 neonates with radiologically confirmed, non-perforated NEC

Patient characteristics	Surgery	Medical management	*p* =	Survived	Died	*p* =	
	(*n* = 84)	(*n* = 107)		(*n* = 161)	(*n* = 30)		
Gender			0.014			0.289	
Male	55 (65.5%)	51 (47.7%)		92 (57.1%)	14 (46.7%)		
Female	29 (34.5%)	56 (52.3%)		69 (42.9%)	16 (53.3%)		
Pregnancy			0.251			0.710	
Single	60 (71.4%)	86 (80.3%)		123 (76.4%)	23 (76.7%)		
Twin	20 (23.8%)	19 (17.8%)		32 (19.9%)	7 (23.3%)		
Triplet	4 (4.8%)	2 (1.9%)		6 (3.7%)	–		
Gestational age			0.003			0.010	
Term	3 (3.6%)	2 (1.9%)		4 (2.5%)	1 (3.3%)		
Preterm (≤ 37 weeks)	25 (29.8%)	58 (54.2%)		77 (47.8%)	6 (20.0%)		
Very preterm (≤ 28 weeks)	56 (66.6%)	47 (43.9%)		80 (49.7%)	23 (76.7%)		
Birth weight			0.112			0.003	
Normal	5 (5.9%)	6 (5.6%)		10 (6.2%)	1 (3.3%)		
Low birth weight (≤ 2500 g)	14 (16.7%)	22 (20.6%)		33 (20.5%)	3 (10.0%)		
Very low birth weight (≤ 1500 g)	13 (15.5%)	30 (28.0%)		42 (26.1%)	1 (3.3%)		
Extremely low birth weight (≤ 1000 g)	52 (61.9%)	49 (45.8%)		76 (47.2%)	25 (83.4%)		

### Predictive value of CRP/ALB ratio for surgery and mortality

**Fig. 1:**
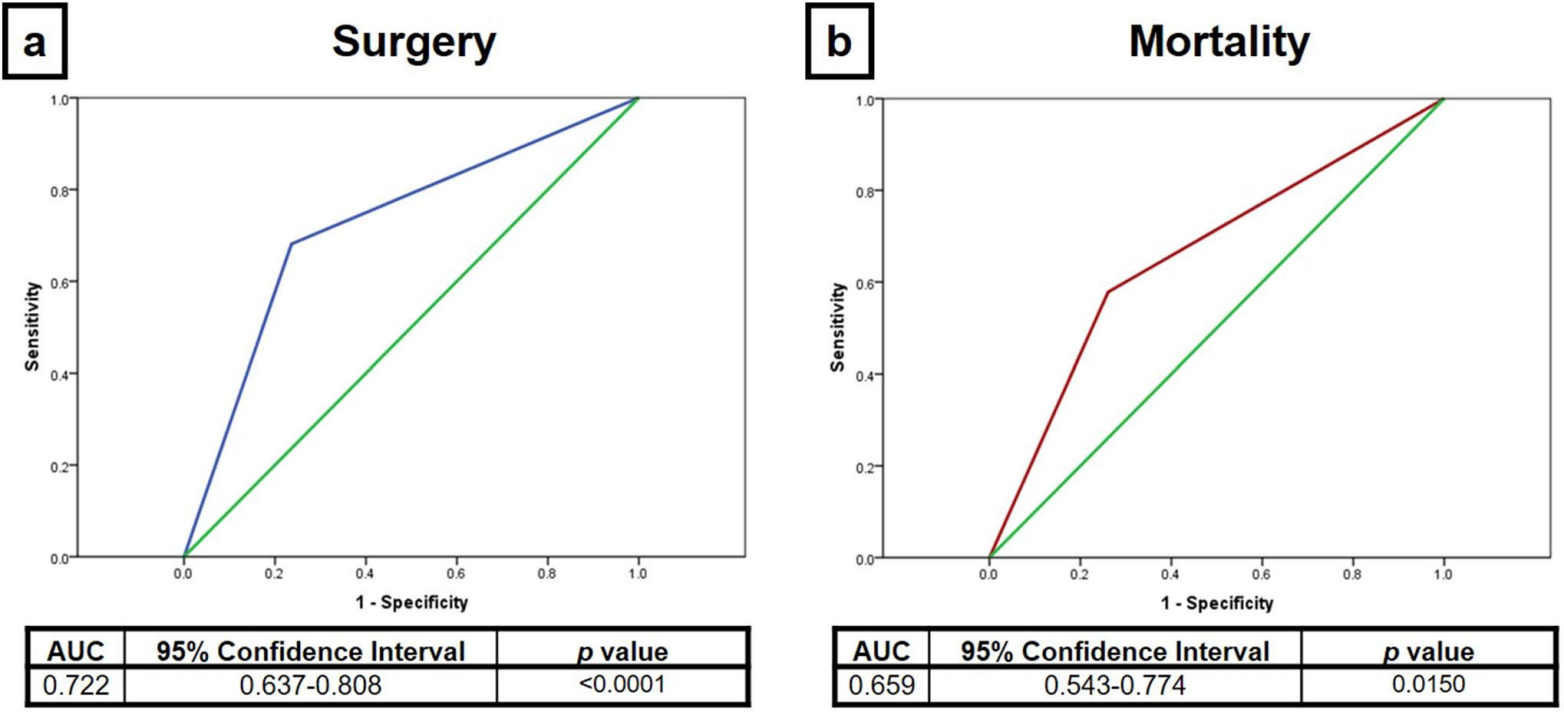
Predictive ability of CRP/ALB ratio of ≥ 3 ratio for (**a**) surgery on day 3 and (**b**) mortality on day 2 in neonates with radiologically confirmed, non-perforated NEC (The green line is a reference line when AUC = 0.5, which would suggest the predictive value is 50%. By comparison, the blue (**a**) and red (**b**) lines represent the AUCs for predicting surgery and mortality, respectively)

Bivariate analysis comparing sensitivity and specificity of different CRP/ALB ratios in predicting surgery and mortality was calculated (Table 2). A serum CRP/ALB ratio of ≥ 2 on day 1 of NEC diagnosis has a reasonably good predictive value for surgical intervention with a sensitivity of 43.9% (95% CI 32.6–55.9) and a specificity of 76.1% (95% CI 66.4–83.6). Sensitivity levels and AUCs increase as medical management of NEC continues from day 1 to 3. The CRP/ALB ratio of ≥ 3 on day 3 had the best predictive ability at sensitivity of 76.4% (95% CI 63.7–85.6) and specificity of 71.9% (95% CI 62.2–79.9) in predicting surgery for NEC with a AUC of 0.72 [(95% CI 0.64–0.81); *p* < 0.0001] (Fig. 1a).

**Table 2:** Sensitivity, specificity and AUC of CRP/ALB ratios in predicting surgery and mortality in neonates with radiologically confirmed, non-perforated NEC

Outcomes	CRP/ALB ratio	Sensitivity (95% CI)	Specificity (95% CI)	AUC (95% CI)	*p* =
Surgery	Day 1 (*n* = 158)				
	CRP/ALB ratio ≥ 2	43.9 (32.6–55.9)	76.1 (66.4–83.6)	0.60 (0.51–0.69)	0.032
	CRP/ALB ratio ≥ 2.5	43.9 (32.6–55.9)	76.1 (66.4–83.6)	0.60 (0.51–0.69)	0.032
	CRP/ALB ratio ≥ 3	39.4 (28.5–51.5)	78.3 (68.8–85.5)	0.59 (0.50–0.68)	0.059
	Day 2 (*n* = 170)				
	CRP/ALB ratio ≥ 2	78.4 (67.7–86.2)	56.3 (46.3–65.7)	0.67 (0.59–0.76)	< 0.001
	CRP/ALB ratio ≥ 2.5	75.7 (53.6–72.5)	63.5 (53.6–72.5)	0.70 (0.62–0.78)	< 0.001
	CRP/ALB ratio ≥ 3	70.3 (59.1–79.5)	68.1 (58.0–76.8)	0.71 (0.63–0.79)	< 0.001
	Day 3 (*n* = 146)				
	CRP/ALB ratio ≥ 2	81.2 (69.7–89.8)	50.6 (40.5–60.6)	0.66 (0.57–0.75)	0.001
	CRP/ALB ratio ≥ 2.5	80.0 (67.6–88.5)	60.4 (50.2–69.9)	0.70 (0.62–0.79)	< 0.001
	CRP/ALB ratio ≥ 3	76.4 (63.7–85.6)	71.9 (62.2–79.9)	0.72 (0.64–0.81)	< 0.001
Mortality	Day 1 (*n* = 158)				
	CRP/ALB ratio ≥ 2	31.8 (16.4–52.7)	67.7 (59.4–74.9)	0.50 (0.37–0.63)	0.968
	CRP/ALB ratio ≥ 2.5	31.8 (16.4–52.7)	67.7 (59.4–74.9)	0.50 (0.37–0.63)	0.968
	CRP/ALB ratio ≥ 3	31.8 (16.4–52-7)	57.8 (49.7–65.5)	0.52 (0.38–0.65)	0.813
	Day 2 (*n* = 170)				
	CRP/ALB ratio ≥ 2	78.3 (58.1–90.3)	44.2 (36.4–52.3)	0.61 (0.50–0.73)	0.083
	CRP/ALB ratio ≥ 2.5	73.9 (53.5–87.5)	49.7 (41.7–57.7)	0.62 (0.50–0.74)	0.069
	CRP/ALB ratio ≥ 3	73.9 (53.3–87.5)	71.3 (63.2–78.3)	0.66 (0.54–0.77)	0.015
	Day3 (*n* = 146)				
	CRP/ALB ratio ≥ 2	76.5 (52.7–90.5)	40.3 (32.2–48.9)	0.58 (0.45–0.72)	0.262
	CRP/ALB ratio ≥ 2.5	76.5 (52.7–90.5)	48.1 (39.6–56.6)	0.62 (0.49–0.76)	0.101
	CRP/ALB ratio ≥ 3	70.6 (46.9–86.7)	54.3 (45.7–62.6)	0.62 (0.49–0.76)	0.096

A serum CRP/ALB ratio of ≥ 3 on day 2 of NEC diagnosis had the highest predictive value for mortality with a sensitivity of 73.9% (95% CI 53.3–87.5), specificity of 71.3% (95% CI 63.2–78.3) and AUC of 0.66 (0.54–0.77); *p* = 0.0150 (Fig. 1b).

## Discussion

In this study, neonates with radiologically confirmed, non-perforated NEC who underwent surgery reflected previously well-established findings in NEC research showing correlation between surgery, male gender and gestational age ≤ 28 weeks. Within this group of patients, we have demonstrated that a CRP/ALB ratio of ≥ 2 early in the disease process (i.e. day 1–3) correlates with surgical intervention.

CRP is an acute phase protein, which will be raised in conditions that provoke a systemic inflammatory response syndrome (SIRS). This has been noted clinically and CRP is often part of the daily tests to monitor neonates for NEC disease progression [11]. A raised CRP in clinically deteriorating NEC cases has been demonstrated [12, 13]. Furthermore, a low serum ALB level has been associated with poor clinical outcomes in critically ill children [14, 15]. Hypoalbuminemia in NEC has recently been identified as a novel marker for disease progression [16].

The significance of the CRP/ALB ratio has previously been reported in a variety of adult conditions [8, 9, 17]. It has been shown that a higher CRP/ALB ratio is an independent prognostic factor in conditions as diverse as chronic kidney disease [18], lung cancer [19, 20] and severe sepsis [9]. The CRP/ALB ratio has also been identified to be an independent predictor of mortality in critically ill or septic adults [21, 22]. There are some comparable research findings reported in pediatrics. In a prospective observational study of 100 critically ill children on a pediatric intensive care unit (PICU), a high CRP/ALB ratio predicted an unfavorable prognosis with adverse outcome (i.e. organ failure and/or death) [10]. Another study looking at 178 pediatric patients used the CRP/ALB ratio to risk stratify children admitted to a PICU [23]. The CRP/ALB ratio within 24 h of PICU admission was utilized. Patients requiring either ventilator or inotropic support had a significantly higher median CRP/ALB ratio. In addition, the CRP/ALB ratio was significantly higher in non-survivors compared with survivors (18.6 *vs.* 4.65; *p* < 0.0001), further indicating that the CRP/ALB ratio at PICU admission is an independent predictor of mortality.

To date, there has only been one single-center study that analyzed the CRP/ALB ratio in neonates. Yang et al. [24] investigated the use of the CRP/ALB ratio in premature infants with perinatal infection. They demonstrated that the ratio is useful for detection of early infection and suggested this as a valuable tool to rationalize the use of antibiotics. A systemic infection in a neonate will trigger a systemic inflammatory response, which is reflected in high serum CRP and low serum albumin levels, and this accounts for these findings. Similarly, although NEC is a disease that originates in the gastrointestinal tract, it has the ability to impact the whole neonate, potentially leading to SIRS. As bowel is progressively compromised during the evolving disease process, the impact of SIRS is reflected in the neonates’ changing physiology and subsequent laboratory serum findings.

Our study has a number of limitations. It was retrospective in nature with a limited number of patients and thus dependent on individually written chart records. Therefore, not reported confounding factors could not necessarily be ruled out, which may have caused a potential result bias. Within single-center studies, there is an overall bias towards how NEC assessment and treatment is carried out. In our center, there are several consultant pediatric surgeons, all operating on and managing neonates with NEC. As a result, the decision-making process is different for each surgeon. In particular, the timing of surgery in a neonate who is medically managed for NEC is very subjective, and hence variable. For example, there are some instances when this variability is uniformly minimized, e.g. in neonates with evidence of bowel perforation.

## Conclusions

To our knowledge, this is the first study analyzing the CRP/ALB ratio specifically in NEC. Our observations showed that a CRP/ALB ratio of ≥ 2 on day 1 has already a reasonably good predictive ability for surgical intervention in neonates with radiologically confirmed, non-perforated NEC. However, a CRP/ALB ratio of ≥ 3 on day 3 had the highest predictive value for surgery in our cohort. This finding may be utilized to triage neonates for transfer to specialist neonatal surgical centers, which would optimize surgical cot utilization. Our study also demonstrated the predicative ability of the CRP/ALB ratio of ≥ 3 on day 2 in view of mortality. This could be used as an additional criterion to guide parental counselling for NEC. Nevertheless, we need to aim for a more standardized decision-making and surgery in NEC. Therefore, we propose a prospective, multicenter study that would enable validation of our results in a larger cohort of neonates with NEC. This would potentially provide a simple, easily accessible and inexpensive tool to provide prediction of surgery and mortality in NEC.
